# Iron Active Center Coordination Reconstruction in Iron Carbide Modified on Porous Carbon for Superior Overall Water Splitting

**DOI:** 10.1002/advs.202401455

**Published:** 2024-04-24

**Authors:** Wenxin Guo, Jinlong Li, Dong‐Feng Chai, Dongxuan Guo, Guozhe Sui, Yue Li, Dan Luo, Lichao Tan

**Affiliations:** ^1^ College of Chemistry and Chemical Engineering Key Laboratory of Fine Chemicals of College of Heilongjiang Province Qiqihar University Qiqihar 161006 China; ^2^ School of Polymer Science & Engineering Qingdao University of Science & Technology Qingdao 266000 China; ^3^ Department of Chemical Engineering University of Waterloo Waterloo ON N2L 3G1 Canada; ^4^ Institute of Carbon Neutrality Zhejiang Wanli University Ningbo 315100 China

**Keywords:** biomass, coordination reconstruction, Fe_3_C, liquid nitrogen quenching, overall water splitting

## Abstract

In this work, a novel liquid nitrogen quenching strategy is engineered to fulfill iron active center coordination reconstruction within iron carbide (Fe_3_C) modified on biomass‐derived nitrogen‐doped porous carbon (NC) for initiating rapid hydrogen and oxygen evolution, where the chrysanthemum tea (elm seeds, corn leaves, and shaddock peel, etc.) is treated as biomass carbon source within Fe_3_C and NC. Moreover, the original thermodynamic stability is changed through the corresponding force generated by liquid nitrogen quenching and the phase transformation is induced with rich carbon vacancies with the increasing instantaneous temperature drop amplitude. Noteworthy, the optimizing intermediate absorption/desorption is achieved by new phases, Fe coordination, and carbon vacancies. The Fe_3_C/NC‐550 (550 refers to quenching temperature) demonstrates outstanding overpotential for hydrogen evolution reaction (26.3 mV at −10 mA cm^−2^) and oxygen evolution reaction (281.4 mV at 10 mA cm^−2^), favorable overall water splitting activity (1.57 V at 10 mA cm^−2^). Density functional theory (DFT) calculations further confirm that liquid nitrogen quenching treatment can enhance the intrinsic electrocatalytic activity efficiently by optimizing the adsorption free energy of reaction intermediates. Overall, the above results authenticate that liquid nitrogen quenching strategy open up new possibilities for obtaining highly active electrocatalysts for the new generation of green energy conversion systems.

## Graphical abstract

The Fe_3_C/NC‐550 modulated by metal active center coordination reconstruction in this work is developed through novel liquid nitrogen quenching treatment for the first time.

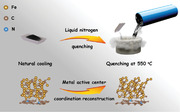

## 1 Introduction

The overconsumed fossil fuels and ever‐growing energy demands have aroused extensive attention from scientific researchers, and a green and efficient energy as alternative is urgently desired.^[^ ^1^ ^]^ Thus, the clean and renewable fuel based on molecular hydrogen (H_2_) is supposed to be an effective solution facing multiple global challenges, including meeting ever‐increasing energy demand, reducing fossil fuel consumption and mitigating climatic deterioration.^[^ ^2^ ^]^ Moreover, hydrogen evolution reaction (HER) featured with accessible reactants, stable output, and feasibility for large‐scale production is supposed as one of the most promising methods among various hydrogen‐generation reaction routes.^[^ ^3^ ^]^ Nowadays, notwithstanding the noble‐metal‐based electrocatalysts are regarded as the most efficient choices, the limited reserves and high cost have hindered their practical application.^[^ ^4^ ^]^ Accordingly, an emerging catalyst is urgently desired as their substitution.

Transition metal‐based oxides, hydroxides, phosphides, sulfides, selenides, nitride, and carbides have been extensively explored as alternatives to noble metal‐based electrocatalysts. In particular, transition metal‐based carbides with favorable electronic conductivity and desirable electrocatalytic behavior come into consideration.^[^ ^5^ ^]^ Nevertheless, the most studies about transition metal‐based carbides focus on the 2D layered transition metal carbon/nitrides (known as MXene), which delivers obvious flaws, such as environmental unfriendly, complicated process, scarce reserves and high cost.^[^ ^6^ ^]^ Accordingly, various alternative catalysts based on nonprecious and earth‐abundant iron group transition metal carbides have garnered much attention ascribed to their remarkable catalytic activity. Iron carbide is one type of intermetallic compounds that carbon atoms occupy the interstices between close‐packed iron atoms. According to the Fe─C phase diagram, regulating carbon content could change crystal phase and structure of iron carbides. Furthermore, the cementite Fe_3_C is stable among these different phases from the viewpoint of thermodynamics.^[^ ^7^ ^]^ Interestingly, the pure‐phase iron carbides and iron carbide assembled on the surface of carbon nanomaterials unfold an noticeable performance gap giving that the H atom is adsorbed on the hollow site of three Fe atoms and the top site of C atom, respectively.^[^ ^8^ ^]^ Moreover, the fabrication route of carbides is usually based on the pyrolysis of metal‐based precursors, while the notion introduced by Ostwald that an intractable problem of thermodynamically agglomerate has troubled Fe_3_C.^[^ ^9^ ^]^ Fortunately, biomass is considered as carbon source attributed to the ability of absorb rather than load Fe^3+^, which can prevent Fe_3_C from aggregating during pyrolysis process effectively. Besides, the conductivity ability of Fe_3_C can be significantly enhanced attributed to the abundant nitrogen sources within biomass.^[^ ^10^ ^]^ Therefore, carbides derived from biomass can be engaged as promising catalyst with environmental friendly, facile preparation process, high yield and low cost.

Moreover, a remarkable catalyst is expected to acquire optimal surface chemistry to ensure active centers with exceptional intrinsic activity and rationally designed architecture to endow catalysts with the full exposure of active sites.^[^ ^11^ ^]^ Therefore, vast quantities of strategies have been proposed for improving electrocatalytic behavior, such as hybridizing with conductive supports, doping with guest elements, incorporating carbon vacancies, introducing lattice strain, designing unique nanostructure and inducing metal coordination reconstruction.^[^ ^12^ ^]^ Among various modification methods, metal active center coordination reconstruction is regarded as an advanced technology to modulate the intermediate absorption/desorption deeply, achieve rapid creation and stabilization of active centers for the following long‐time operation, reduce the whole energy consumption during electro‐catalysis, and achieve the overall reaction stability.^[^ ^13^ ^]^ Hitherto, the construction methods of metal active center coordination reconstruction are highly constrained by heteroatom introduction and high temperature calcination. For instance, Cui et al. reported a Ni active center coordination reconstruction achieved by multidimensional modulation of phase transition, iodine coordination, and carbon vacancies.^[^ ^14^ ^]^ Sun et al. proposed phase modulated polymorphic cobalt‐based catalysts with tailorable nitrogen‐metal bonds through a cationic molybdenum‐substitution strategy.^[^ ^15^ ^]^ Hu et al. constructed (Co_x_Fe_1–x_)_3_N with high dispersibility induced by an intermediate phase transition process.^[^ ^9^ ^]^ In this regard, a novel and efficient method is urgently needed to achieve coordination reconstruction precisely. In our unpublished work, the NiFe_2_O_4_ nanosheets synergetic modulated by divalent/trivalent mixed cation vacancies and lattice tensile strain are developed through chemical etching and novel liquid nitrogen quenching treatment, the unit cell lattice parameter of NiFe_2_O_4_ has been expand successfully owing to the instantaneous thermal expansion when NiFe_2_O_4_ with surface high‐temperature (50–350 °C) is subjected to rapid quenching in liquid nitrogen immediately after calcination process, leading to lattice tensile strain on electrocatalysts support‐surface, which reminds us to put forward the hypothesis that the relatively weak forces caused by lower quenching temperature could make the unit cell lattice parameter expand, while the higher quenching temperature (350–750 °C) could generate strong resultant force, the corresponding strong force could destroy the preceding bonds and form new ones, thereby achieving metal active center coordination reconstruction. However, to the best of our knowledge, no related works have been reported on the use of liquid nitrogen quenching treatment to induce metal active center coordination reconstruction precisely.

Inspired by these above considerations, in this work, a novel liquid nitrogen quenching strategy is engineered to achieve iron (Fe) active center coordination reconstruction within iron carbide modified on biomass‐derived nitrogen (N)‐doped porous carbon (NC) layers, in which the chrysanthemum tea (elm seeds, corn leaves, and shaddock peels, etc.) is designed to serve as carbon source for constructing Fe_3_C and NC, the earth‐abundant biomass precursors in this approach are both easily obtained and environmentally friendly, which makes the material suitable for large‐scale production. Meanwhile, the original thermodynamic stability is changed through the corresponding force generated by liquid nitrogen quenching and the transformation from Fe_3_C (Cohenite, syn PDF#97‐001‐6593) to Fe_3_C (Iron Carbide PDF#97‐016‐7667) is induced with rich carbon vacancies with the increasing instantaneous temperature drop amplitude. The optimizing intermediate absorption/desorption is achieved by new phases, Fe coordination, and carbon vacancies. Density functional theory (DFT) calculations further confirm that liquid nitrogen quenching treatment can enhance intrinsic electrocatalytic activity efficiently by optimizing the adsorption free energy of reaction intermediates. Overall, the novel liquid nitrogen quenching strategy opens up a new avenue to obtain high‐activity electrocatalysts for a new generation of green energy conversion system.

## 2 Results and Discussion

**Scheme 1:**
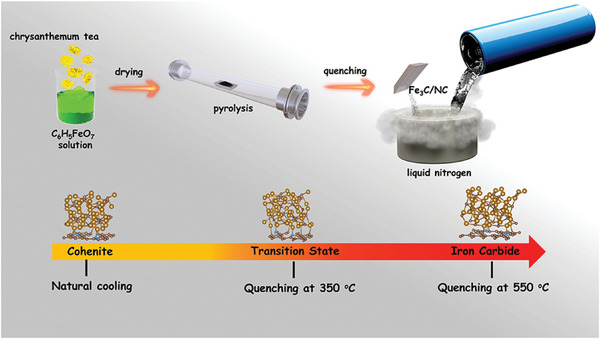
The preparation scheme of Fe_3_C/NC‐X.

Fe active center coordination reconstruction within iron carbide modified on biomass‐derived N‐doped porous carbon layers (Fe_3_C/NC‐550) is obtained by pyrolysis followed by liquid nitrogen quenching process by employing environmentally friendly precursors of chrysanthemum tea and C_6_H_5_FeO_7_. A detailed route of converting chrysanthemum tea and C_6_H_5_FeO_7_ into Fe_3_C/NC‐X (X stands for quenching temperature) is schematically illustrated in **Scheme** **1**. Initially, the fresh chrysanthemum tea is immersed into aqueous solution containing C_6_H_5_FeO_7_, and the dried chrysanthemum tea is subjected to high temperature pyrolysis, C_6_H_5_FeO_7_ could be decomposed and generates α‐Fe nanoparticles. Meanwhile, the carbon‐containing gases and amorphous carbon (decomposed from chrysanthemum tea) diffuse into the as‐obtained α‐Fe particles and release carbon atoms, which are then graphitized to form Fe_3_C nanoparticles modified on porous carbon, the deposited carbon could act as support to anchor Fe_3_C nanoparticles with high dispersion.^[^ ^16^ ^]^ Subsequently, the obtained Fe_3_C/NC with various surface high temperature is executed to rapid quenching in liquid nitrogen (−196 °C) immediately after pyrolysis treatment, achieving an ultrahigh cooling rate (246–946 °C s^−1^), the original crystal structure of iron carbide is destroyed due to the resultant force generated by instantaneous cooling, thereby achieving metal active center coordination reconstruction.

**Figure 1:**
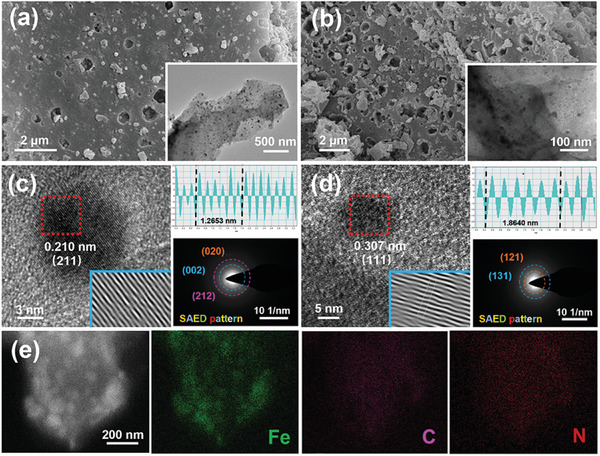
SEM images of Fe_3_C/NC (a) and Fe_3_C/NC‐550 (b); insert charts: TEM images of Fe_3_C/NC and Fe_3_C/NC‐550; HRTEM images of Fe_3_C/NC (c) and Fe_3_C/NC‐550 (d); insert charts: FFT, IFFT and SAED images of Fe_3_C/NC and Fe_3_C/NC‐550; TEM mapping of Fe_3_C/NC‐550 (e).

**Figure 2:**
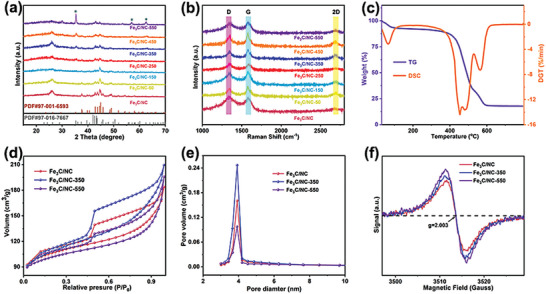
XRD spectrum of Fe_3_C/NC, Fe_3_C/NC‐50, Fe_3_C/NC‐150, Fe_3_C/NC‐250, Fe_3_C/NC‐350, Fe_3_C/NC‐450 and Fe_3_C/NC‐550 (a); Raman spectrum of Fe_3_C/NC, Fe_3_C/NC‐50, Fe_3_C/NC‐150, Fe_3_C/NC‐250, Fe_3_C/NC‐350, Fe_3_C/NC‐450 and Fe_3_C/NC‐550 (b); TG and DSC spectra of Fe_3_C/NC‐550 (c); N_2_ adsorption–desorption isotherms of Fe_3_C/NC, Fe_3_C/NC‐350 and Fe_3_C/NC‐550 (d); The pore diameter distribution of Fe_3_C/NC, Fe_3_C/NC‐350 and Fe_3_C/NC‐550 (e); EPR spectrum of Fe_3_C/NC, Fe_3_C/NC‐350 and Fe_3_C/NC‐550 (f).

The morphology and microstructure of Fe_3_C/NC, Fe_3_C/NC‐350, and Fe_3_C/NC‐550 are explored by scanning electron microscopy (SEM) and transmission electron microscopy (TEM), respectively. The dispersed nanoparticles scattered on sheet‐like nanostructure have been illustrated clearly (**Figure** **1**a,b), demonstrating the effective embedment of Fe_3_C nanoparticles on the surface of biomass‐derived N‐doped carbon, which could facilitate the exposure of rich electrochemical active sites.^[^ ^17^ ^]^ Meanwhile, Fe_3_C nanoparticles hold strong coupling with N‐doped carbon with π–π interactions between Fe_3_C nanoparticles and N‐doped carbon, which is beneficial to electrocatalytic stability.^[^ ^18^ ^]^ Fe_3_C/NC‐350 reveals the same phenomenon as well (Figure S1, Supporting Information). Furthermore, the distinct lattice fringes are demonstrated in high‐resolution TEM (HR‐TEM) images (**Figure** **2**c,d), revealing two types of iron carbide before and after liquid nitrogen quenching treatment. The detected interplanar spacing distance of 0.210 and 0.307 nm is corresponded to the (211) and (111) crystal planes of Fe_3_C (Cohenite, syn PDF#97‐001‐6593) and Fe_3_C (Iron Carbide PDF#97‐016‐7667) benefiting from the phase transformation of the as‐obtained catalysts after liquid nitrogen quenching treatment. Meanwhile, the detailed lattice parameters are obtained by fast Fourier transform (FFT) and inverse fast Fourier transform (IFFT). Furthermore, the selected‐area electron diffraction (SAED) patterns of Fe_3_C/NC and Fe_3_C/NC‐550 samples confirm the phase transform of Fe_3_C successfully. Additionally, the TEM mapping images demonstrate that C and N elements are uniform. Notably, Fe elements reveal a distinct blocky distribution attributed to the dispersed iron carbide nanoparticles (Figure 2e). Moreover, the SEM and EDS mapping of Fe_3_C/NC‐550 are displayed in Figure S2 (Supporting Information).

X‐ray diffraction (XRD) analysis is adopted to further explore crystalline structure and confirm the phase evolution of the obtained Fe_3_C modified on biomass‐derived N‐doped porous carbon layers (Figure 2a; Figure S3, Supporting Information). The carbon diffraction peak is relatively weak attributed to the formation of Fe_3_C nanoparticles on chrysanthemum tea‐derived carbon. Meanwhile, the increasing nitrogen quenching temperature results in the phase transformation from Fe_3_C (Cohenite, syn PDF#97‐001‐6593) to Fe_3_C (Iron Carbide PDF#97‐016‐7667). Significantly, two types phases are clearly identified at the nitrogen quenching temperature of 350 °C. This phenomenon confirms that the phase transformation is a gradual process tied with liquid nitrogen quenching temperature closely. To further clarify the role of liquid nitrogen quenching treatment as a phase stabilizer, the reference sample (Fe_3_C/NC) is synthesized without liquid nitrogen quenching treatment, in which only Fe_3_C (Cohenite, syn PDF#97‐001‐6593) is obtained. Notably, a reduction atmosphere is critical for forming carbide phase, only iron oxides and carbon could be obtained without hydrogen atmosphere. In addition, various earth‐abundant biomass precursors such as elm seeds, corn leaves, and shaddock peels have been applied to serve as carbon source within iron carbide and N‐doped porous carbon layers successfully. The XRD patterns (Figures S4–S6, Supporting Information) exhibit similar results of phase transformation, indicating the applicability of the proposed strategy.

Furthermore, the Raman spectrum is applied to investigate the structural characteristics of the as‐obtained electrocatalysts (Figure 2b). As a consequence, D band (sp^3^ carbon) and G band (sp^2^ carbon) situate at 1350 and 1590 cm^−1^, respectively. In particular, graphitic carbon is beneficial to electrical conductivity, while defective carbon would provide abundant active sites. The moderate ID/IG ratio of as‐obtained catalysts indicates a proper balance between the degree of graphitization and the carbon defects, demonstrating promising potential to enhance electrocatalytic performance.^[^ ^19^ ^]^ Thermogravimetry (TG) analysis (Figure 2c) further determines the accurate contents of Fe_3_C in Fe_3_C/NC‐550, the negligible weight loss below 100 °C is related with the loss of absorbed water. When the temperature rises from 100 to 600 °C, carbon components (i.e., amorphous carbon and graphitic carbon) and Fe_3_C combusts to CO_2_ and Fe_2_O_3_, respectively, accelerating the weight loss. Thus, the Fe_3_C content in Fe_3_C/NC‐550 is calculated to be 10.2%.^[^ ^20^ ^]^ Brunauer–Emmett–Teller (BET) surface area and pore size distribution are measured by N_2_ adsorption–desorption analysis, the characteristic of mesoporous materials is observed for Fe_3_C/NC, Fe_3_C/NC‐350 and Fe_3_C/NC‐550, and the high specific surface area of 319.9 m^2^ g^−1^ is obtained for Fe_3_C/NC‐550 sample (Figure 2d), and a typical mesoporous structure can be obtained from the prepared electrocatalysts (Figure 2e). Electron paramagnetic resonance (EPR) is conducted to explore the electron spin state of Fe atomic orbitals, and Fe_3_C/NC‐550 owns the highest signal intensity compared to the other samples (Figure 2f), connecting with more unpaired electrons from carbon vacancies owing to the fact that strong resultant force generated by liquid nitrogen quenching destroy the preceding bonds.^[^ ^14^ ^]^ Quenching temperature could regulate electron spins, and Fe_3_C/NC‐550 possess the optimal electronic structure among the other catalysts.

**Figure 3:**
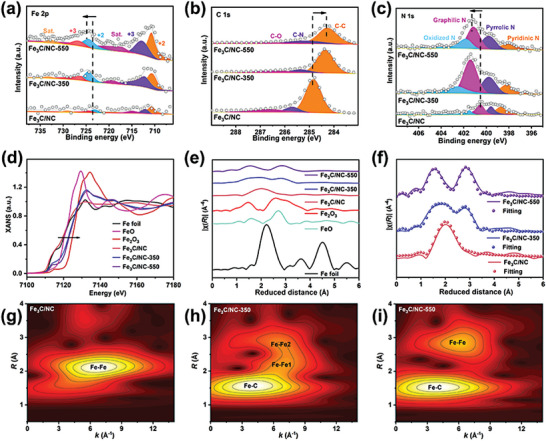
XPS spectra of Fe_3_C/NC, Fe_3_C/NC‐350 and Fe_3_C/NC‐550: Fe 2p (a), C 1s (b), and N 1s (c); XANES spectra of the Fe K‐edge (d); Fourier transform of EXAFS spectra at the Fe K‐edge (e); Fitting results for Fe FT‐EXAFS spectra (f); Wavelet transformed plots for the k^3^‐weighted EXAFS signals of the Fe K‐edge of Fe_3_C/NC (g), Fe_3_C/NC‐350 (h), and Fe_3_C/NC‐550 (i).

The electronic structure and chemical elements are conducted by X‐ray photoelectron spectroscopy (XPS). The XPS survey spectra of Fe_3_C/NC, Fe_3_C/NC‐350, and Fe_3_C/NC‐550 ensures the coexistence of Fe, C and N elements (Figure S7, Supporting Information). The high‐resolution spectra for each element are recorded to examine the presence of different chemical states. As illustrated in **Figure** **3**a, a high binding energy shift of the Fe─C bond is observed in Fe_3_C/NC‐550 compared to Fe_3_C/NC and Fe_3_C/NC‐350, ascribed to the electron‐lack of Fe atoms in the new phase. The increase in the electron density of the C site is known from the shift of the C peak to lower binding energy (Figure 3b). Besides, N peak in Fe_3_C/NC‐550 delivers a higher binding energy shift compared to Fe_3_C/NC and Fe_3_C/NC‐350, implying a stronger coupling between Fe and N (Figure 3c). Generally, pyridinic N is proposed to act a momentous role in enhancing the electrocatalytic activity. As a consequence, electrons are gradually transferred from Fe/N to C under the effect of the liquid nitrogen quenching temperature. X‐ray absorption near‐edge structures (XANES) and X‐ray absorption fine structures (EXAFS) spectra are utilized to unveil chemical states and coordination environment. As shown in Figure 3d, the valence state of Fe in Fe_3_C/NC, Fe_3_C/NC‐350, and Fe_3_C/NC‐550 is between FeO and Fe_2_O_3_. After the liquid nitrogen quenching induced formation of Fe_3_C/NC‐550, the increased absorption edge of Fe in agreement with the XPS results. The R‐space obtained by Fourier transform and extended XANES oscillation functions k2 𝜒(k) reveals Fe coordination clearly. The appearance of new bond and the disappearance of old bond in Fe_3_C/NC‐550 are indicative of the production of new iron active center coordination structure compared to Fe_3_C/NC and Fe_3_C/NC‐350 (Figure 3e). The Fe_3_C/NC, Fe_3_C/NC‐350, and Fe_3_C/NC‐550 plots reveal the markedly different k space oscillation (Figure S8, Supporting Information), suggesting that the liquid nitrogen quenching can significantly regulate the local atomic environment of Fe_3_C/NC‐550.^[^ ^21^ ^]^ Furthermore, the Fe coordination number and interatomic bonding distance are investigated by EXAFS fitting analysis. The fitting results for Fe FT‐EXAFS spectra of Fe_3_C/NC, Fe_3_C/NC‐350, and Fe_3_C/NC‐550 are declared visually in Figure 3f and Table S1 (Supporting Information), further confirms that the Fe‐centered coordination environment has been completely transformed. The coordination numbers of Fe─Fe and Fe─C for Fe_3_C/NC‐550 are calculated as 3.96 ± 0.38 and 2.63 ± 0.19 at distances of 3.063 ± 0.036 Å and 1.991 ± 0.096 Å, respectively, which are different from that of the Fe_3_C/NC (coordination numbers of Fe─Fe and Fe─C are calculated as 3.32 ± 0.12 and 3.04 ± 0.17 at distances of 2.456 ± 0.023 Å and 2.023 ± 0.064 Å). The dramatic change in coordination number and interatomic bonding distance is conclusive evidence to confirm the coordination environment reconstruction.^[^ ^22^ ^]^ Moreover, the coordination environment of Fe is well regulated with a higher Fe─Fe/C coordination number, which triggers more optimized adsorption strength between active sites and reaction intermediates.^[^ ^23^ ^]^ The wavelet transform (WT) EXAFS contour map arrays the appearance of new bond and the disappearance of old bond (Figure 3g–i), confirming the Fe active center coordination reconstruction.^[^ ^24^ ^]^ As a comparison, the references of Fe foil and Fe_2_O_3_ are displayed in Figure S9 (Supporting Information). The above characterization provides evidence for the Fe active center coordination reconstruction.

**Figure 4:**
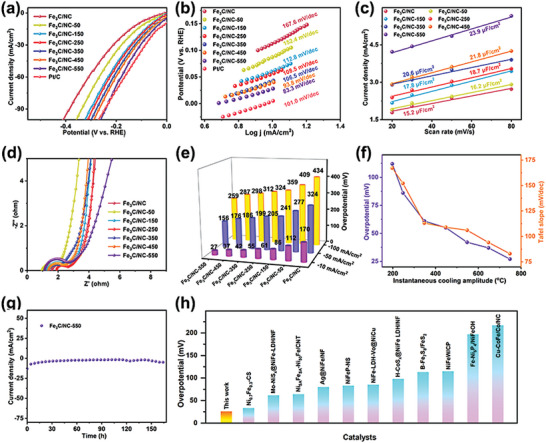
LSV curves (a) and Tafel slopes (b) of Pt/C, Fe_3_C/NC, Fe_3_C/NC‐50, Fe_3_C/NC‐150, Fe_3_C/NC‐250, Fe_3_C/NC‐350, Fe_3_C/NC‐450 and Fe_3_C/NC‐550; ECSA (c) and EIS (d) of Fe_3_C/NC, Fe_3_C/NC‐50, Fe_3_C/NC‐150, Fe_3_C/NC‐250, Fe_3_C/NC‐350, Fe_3_C/NC‐450 and Fe_3_C/NC‐550; The overpotential of Pt/C, Fe_3_C/NC, Fe_3_C/NC‐50, Fe_3_C/NC‐150, Fe_3_C/NC‐250, Fe_3_C/NC‐350, Fe_3_C/NC‐450 and Fe_3_C/NC‐550 at −10, −50, and −100 mA cm^−2^ (e); The overpotential and Tafel slopes of Fe_3_C/NC at different instantaneous cooling amplitude (f); The *i*–*t* curve of Fe_3_C/NC‐550 for HER at 10 mA cm^−2^ (g); The HER performance of other Fe‐based electrocatalysts (h).

The electrocatalytic activity of Fe_3_C/NC, Fe_3_C/NC‐50, Fe_3_C/NC‐150, Fe_3_C/NC‐250, Fe_3_C/NC‐350, Fe_3_C/NC‐450, Fe_3_C/NC‐550, Fe_3_C/NC‐650, Fe_3_C/NC‐750, and Pt/C for HER is evaluated in 1.0 m KOH solution at room temperature through linear sweep voltammetry (LSV) curves in a representative three‐electrode system. As manifested in **Figures** **4**a and S10 (Supporting Information), the overpotential of Fe_3_C/NC‐550 is merely 26.3 mV to drive a current density of −10 mA cm^−2^, which is superior to those of Fe_3_C/NC (111.3 mV), Fe_3_C/NC‐50 (87.6 mV), Fe_3_C/NC‐150 (60.9 mV), Fe_3_C/NC‐250 (56.5 mV), Fe_3_C/NC‐350 (42.9 mV), Fe_3_C/NC‐450 (38.5 mV), Fe_3_C/NC‐650 (27.8 mV), and Fe_3_C/NC‐750 (30.4 mV), respectively. Furthermore, it is remarkable fact that the HER activity of Fe_3_C/NC‐550 surpasses most of the recently reported Fe‐based electrocatalysts (Figure 4h).^[^ ^25^ ^]^ Meanwhile, Fe_3_C/NC‐550 still manifests the lowest overpotential of 156.9 and 259.3 mV at −50 and −100 mA cm^−2^ (Figure 4e), ascribed to the coordination reconstruction of Fe active center. Especially, electrocatalytic hydrogen evolution activity boosts with the in‐depth degree of coordination reconstruction caused by the strong resultant force at increasing quenching temperature, as shown in Figure 4f. For comparison, upon employing the novel pyrolysis and liquid nitrogen quenching route, Fe active center coordination reconstruction within Fe_3_C/NC prepared by other biomass‐based materials such as elm seeds, corn leaves, and shaddock peel has been realized successfully and the obtained electrocatalysts deliver the outstanding overpotential of 51.2, 55.2, and 62.0 mV (Figure S11, Supporting Information), respectively, suggesting the universality of the proposed strategy. In addition, the reaction kinetics of the electrocatalysts is estimated by Tafel slope and electrochemical impedance (EIS), respectively. In Figure 4b, Fe_3_C/NC‐550 carries the smallest Tafel slope of 83.3 mV dec^−1^, which is much lower than those of the corresponding Fe_3_C/NC (167.6 mV dec^−1^), Fe_3_C/NC‐50 (152.4 mV dec^−1^), Fe_3_C/NC‐150 (112.8 mV dec^−1^), Fe_3_C/NC‐250 (108.5 mV dec^−1^), Fe_3_C/NC‐350 (106.5 mV dec^−1^) and Fe_3_C/NC‐450 (93.8 mV dec^−1^), respectively. The smallest Tafel slope of Fe_3_C/NC‐550 testifies the highest transfer coefficient and fastest HER kinetics profited from the reconstruction of the coordination environment. Furthermore, as shown in Figure 4d, these catalysts display extremely low resistance by virtue of the remarkable electrical conductivity provided by the nitrogen‐doped carbon. Moreover, the exposure of active sites is reflected by electrochemical surface area (ECSA), which is evaluated by the double‐layer capacitance (*C*_dl_). As indicated in Figure 4c, the *C*_dl_ of Fe_3_C/NC, Fe_3_C/NC‐50, Fe_3_C/NC‐150, Fe_3_C/NC‐250, Fe_3_C/NC‐350, Fe_3_C/NC‐450, and Fe_3_C/NC‐550 is 15.2, 16.2, 17.8, 18.7, 20.6, 21.8, and 23.9 µF cm^−2^, respectively, indicating the highest density of catalytical active sites of Fe_3_C/NC‐550. Figure S12 (Supporting Information) displays the LSV curves of as‐obtained catalysts after normalizing the current density by ECSA. As concerns of potential application raise, the electrochemical stability and durability of Fe_3_C/NC‐550 are examined through a typical amperometric *i*–*t* measurement at the current density of −10 mA cm^−2^. As indicated in Figure 4g, the stability of Fe_3_C/NC‐550 in continuous HER is evaluated by amperometric *i*–*t* at a voltage of 1.097 V, and the overpotential has been maintained efficiently after 168 h, demonstrating its prominent stability. The *i*–*t* test at higher current density of −100 mA cm^−2^ are performed for 92 h, and the catalyst possesses a retention rate of more than 75% (Figure S13, Supporting Information).

**Figure 5:**
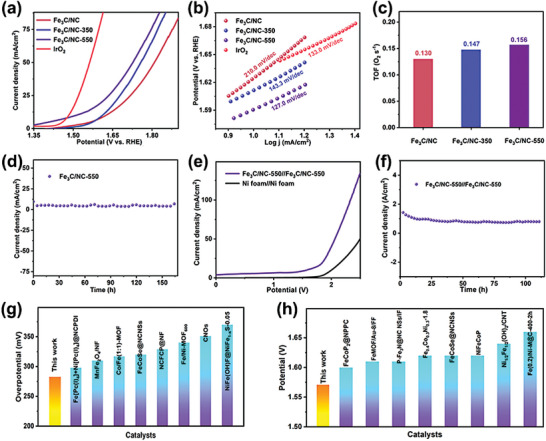
OER LSV curves (a) and Tafel slopes (b) of IrO_2_, Fe_3_C/NC, Fe_3_C/NC‐350 and Fe_3_C/NC‐550; TOF values of Fe_3_C/NC, Fe_3_C/NC‐350 and Fe_3_C/NC‐550 (c); *i*–*t* curve of Fe_3_C/NC‐550 for OER (d); The overall water spitting LSV curve of Fe_3_C/NC‐550//Fe_3_C/NC‐550 (e); *i*–*t* curve of Fe_3_C/NC‐550 for overall water spitting (f); OER (g) and overall water spitting (h) performance for Fe_3_C/NC‐550 and other catalysts.

The OER performance of Fe_3_C/NC, Fe_3_C/NC‐350, Fe_3_C/NC‐550, and IrO_2_ is carried out through a representative three‐electrode equipment in 1.0 m KOH electrolyte with a pure graphite rod as the counter electrode. Especially, Fe_3_C/NC‐550 emerges the wonderful OER performance as expected. As can be seen from **Figure** **5**a, the LSV curves uncover that the as‐synthesized Fe_3_C/NC‐550 electrocatalyst demonstrates an overpotential of merely 281.4 mV at 10 mA cm^−2^ to generate OER current, which is significantly lower than those of Fe_3_C/NC (395.4 mV), and Fe_3_C/NC‐350 (381.4 mV), respectively. Fe_3_C/NC‐550 demonstrates an enhanced OER behavior compared to related reports (Figure 5g).^[^ ^26^ ^]^ Moreover, Fe_3_C/NC‐550 still delivers the best OER performance at the current densities of 50 and 100 mA cm^−2^. As known, the Tafel slope as a key kinetic parameter is a common method to investigate the rate‐determining step of OER processes. Fe_3_C/NC‐550 manifests a value Tafel slope (127.0 mV dec^−1^), which is lower than Fe_3_C/NC (210.0 mV dec^−1^), and Fe_3_C/NC‐350 (143.3 mV dec^−1^), indicating that Fe_3_C/NC‐550 owns beneficial reaction kinetics (Figure 5b). Afterward, the turnover frequency (TOF) of catalysts is determined to illustrate the intrinsic activity (Figure 5c). The high TOF is obtained for the Fe_3_C/NC‐550 (0.156 s^−1^), prominently exceeding those of Fe_3_C/NC (0.130 s^−1^) and Fe_3_C/NC‐350 (0.147 s^−1^). Simultaneously, Fe_3_C/NC‐550 confirms the long‐term stability via the amperometric *i*–*t* test at 10 mA cm^−2^ in 1.0 m KOH electrolyte, which can be stable for 168 h without obvious decay, declaring its value stability and durability (Figure 5d). Encouraged by the extraordinary activity of Fe_3_C/NC‐550 for HER and OER in alkaline media, an alkaline electrolyzer is assembled with Fe_3_C/NC‐550 serving as both cathode and anode. The device affords the current density of 10 mA cm^−2^ at cell voltages of 1.57 V (Figure 5e) and maintains the majority of performance after 110 h (Figure 5f) in 1.0 m KOH. Besides, the morphology, phase, and valence state of sample are well maintained after cycling tests (Figures S14–S16, Supporting Information). Faraday efficiency can be evaluated for the energy conversion of Fe_3_C/NC‐550//Fe_3_C/NC‐550 cells. Therefore, the molar ratio of H_2_ and O_2_ is calculated during overall water splitting (Figure S17, Supporting Information). The theoretical data are basically consistent with the experimental data, which proves that the Fe_3_C/NC‐550//Fe_3_C/NC‐550 electrocatalytic reaction process high energy conversion rate. In addition, the overall water splitting performance of Fe_3_C/NC‐550 precedes those of recently reported Fe‐based electrocatalysts (Figure 5h).^[^ ^27^ ^]^

Overall, the resultant Fe_3_C/NC‐550 catalyst delivers a favorable adsorption/desorption kinetics for achieving rapid and stable HER and OER, as evidenced by the lowest overpotential at −10 and 10 mA cm^−2^, respectively. By experimental result characterizations, the underlying promotion mechanism is revealed and summarized as follows: i) Metal active center coordination reconstruction could optimize the intermediates absorption/desorption deeply, achieve rapid creation and stabilization of active centers for following long‐time operation, reduce the whole energy consumption during the electro‐catalysis, and enhance the overall reaction stability; ii) Carbon vacancies could modulate the surface electronic structure of the electrocatalyst, act as charge carrier acceptors and free electron compensating centers to promote the reaction kinetics; iii) A carbide‐loaded carbon layer could boost electrons transfer within material, and the nitrogen further enhances electrical conductivity, meanwhile, in situ carbides can effectively avoid aggregation of material to increase electrocatalytic active sites.

**Figure 6:**
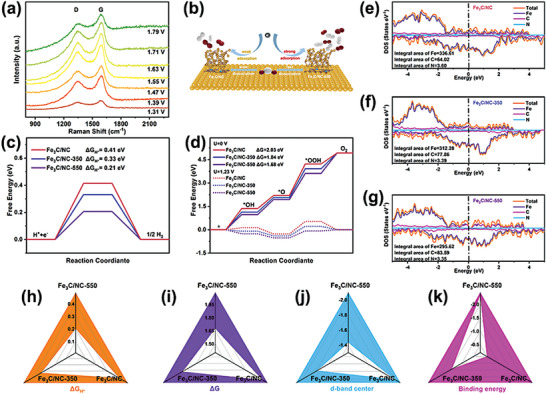
In situ Raman spectra of Fe_3_C/NC‐550 at different applied potentials (a); The schematic diagram of proposed reaction mechanism for Fe_3_C/NC and Fe_3_C/NC‐550 (b); Free energy diagram of HER (c) and OER (d) for Fe_3_C/NC, Fe_3_C/NC‐350 and Fe_3_C/NC‐550; Density of states for Fe_3_C/NC (e), Fe_3_C/NC‐350 (f) and Fe_3_C/NC‐550 (g); Radar plots of Fe_3_C/NC, Fe_3_C/NC‐350 and Fe_3_C/NC‐550 correspond to ΔG_H*_ (h), ΔG (i), d‐band center (j), and binding energy (k).

Further investigation on the relationship between anodic current and the properties of electrode materials is essential for mechanistic understanding and functional design. The in situ Raman spectra at various applied potentials illustrate that the G band intensity of Fe_3_C/NC‐550 increase as the application potentials, which means the increasing graphitization degree, resulting in enhanced conductivity (**Figure** **6**a). Density functional theory (DFT) calculation is utilized to elucidate Fe active center coordination reconstruction and the electrocatalytic activity evaluation. The HER and OER chemisorption processes of Fe_3_C/NC‐550 are illustrated in Figures S18 and S19 (Supporting Information). Figure 6b delivers the schematic diagram of proposed reaction mechanism for Fe_3_C/NC and Fe_3_C/NC‐550. The H adsorption free energy (ΔG_H*_) is explored to evaluate its theoretical catalytic activity. Fe_3_C/NC‐550 offers a ΔG_H*_ of 0.21 eV that is near to 0 eV compared to Fe_3_C/NC (0.41 eV) and Fe_3_C/NC‐350 (0.33 eV), respectively (Figure 6c). The low ΔG_H*_ benefits the HER performance.^[^ ^3a^ ^]^ Whereafter, the Gibbs free energy (ΔG) profile of the OER intermediates is calculated, as compared in Figure 6d. The adsorption of the ^*^OOH intermediate on the reference Fe_3_C/NC catalyst is the rate‐determining step (RDS) with the largest free energy barrier of 2.03 eV without the application of an external potential (U = 0 V). After the coordination reconstruction, the free energy of the RDS significantly drops to 1.68 eV, suggesting that the new coordination environment is beneficial to optimize the intermediate adsorption during OER process and thus lowering the OER overpotential.^[^ ^28^ ^]^ The whole reaction can be readily proceeded when an external potential of 1.23 V is applied. From this viewpoint, the new coordination environment within Fe_3_C/NC‐550 catalyst is critical to enhance the OER catalysis. Furthermore, the continuously distributed density of states (DOS) and abundant electrical states at the Fermi level suggest the favorable conductivity of catalysts (Figure 6e–g). The partial DOS validates a major contribution from Fe atoms toward the total DOS, confirming that the Fe element is the main catalytic active center within Fe_3_C/NC‐550. Noteworthily, the decreased integral area of 295.62/3.35 on Fe/N can be achieved for Fe_3_C/NC‐550, indicating the electron depletion on Fe/N atoms. On the contrary, the increased integral area of 83.59 on C represents the accumulation of electron.^[^ ^29^ ^]^ As expected, the decreased binding energy of XPS is consistent with the theoretically calculated variations of charge density on Fe, C, and N. The above results can be summarized in the following two points: i) Fe_3_C/NC‐550 acquires the optimal Gibbs free energy (both ΔG_H*_ for HER and ΔG for OER), in which the absolute value is much lower than those of Fe_3_C/NC and Fe_3_C/NC‐350, respectively, which ensures a faster adsorption/desorption reaction process. ii) The obtained results confirm that the d‐band center value of the Fe_3_C/NC‐550 gradually approaches 0 eV with the progress of the experimental steps, demonstrating that the theoretical catalytic activity of the electrocatalyst is gradually improved. Visually, the ΔG_H*_ and ΔG of the catalysts are presented in Figure 6h,i, where Fe_3_C/NC‐550 exhibits remarkable advantages compared to the others. Meanwhile, the coordination reconstruction changes the d‐band center approach of the Fermi energy level (Figure 6j; Figure S20, Supporting Information), which makes up for the lack of adsorption energy. Ultimately, the ability of Fe_3_C/NC‐550 catalysis stands out when the binding energy of Fe_3_C and carbon substrates are constructed (Figure 6k).

## 3 Conclusion

In summary, for the first time, a metal active center coordination reconstruction strategy is proposed by introducing liquid nitrogen quenching treatment, resulting in Fe active center coordination reconstruction and carbon vacancies. The metal active center coordination environment is accurately controlled by quenching temperature and fully verified by EPR, XPS and EXAFS techniques. Fe_3_C/NC‐550 demonstrates superior water‐splitting performance compared to most of the reported materials. The overpotential is 26.3 mV for HER at −10 mA cm^−2^ and 281.4 mV for OER at 10 mA cm^−2^. Only 1.57 V is required to drive 10 mA cm^−2^ overall water splitting in an alkaline electrolyzer. Overall, this coordination reconstruction concept and material design method provides new tactics for designing superior electrocatalysts.

## 4 Experimental Section

### Preparation of Fe_3_C/NC

A C_6_H_5_FeO_7_ solution was prepared by dissolving 3 g FeCl_3_, 6 g NaHCO_3_, and 8 g C_6_H_8_O_7_ in 50 mL deionized water. Then, 5 g chrysanthemum tea was immersed into the as‐prepared C_6_H_5_FeO_7_ solution. After the subsequent drying process, the as‐dipped chrysanthemum tea is inserted into the center of a horizontal tube furnace and thermally treated at 750 °C for 10 min with a continuous flow of hydrogen and argon mixed gases to synthesize Fe_3_C/NC.

### Preparation of Fe_3_C/NC‐550

Typically, the obtained Fe_3_C/NC nanosheets with surface high‐temperature of 550 °C are executed to rapid quenching in liquid nitrogen immediately after calcination treatment without natural cooling. As a contrast, Fe_3_C/NC‐50, Fe_3_C/NC‐150, Fe_3_C/NC‐250, Fe_3_C/NC‐350, Fe_3_C/NC‐450, Fe_3_C/NC‐650 and Fe_3_C/NC‐750 followed the similar preparation route when the quenching temperature was 50, 150, 250, 350, 450, 650, and 750 °C, respectively.

## Conflict of Interest

The authors declare no conflict of interest.

## Supporting information

Supporting Information

## Data Availability

The data that support the findings of this study are available from the corresponding author upon reasonable request.
