# Supplementary material for: Iron Active Center Coordination Reconstruction in Iron Carbide Modified on Porous Carbon for Superior Overall Water Splitting
Source: Adv Sci (Weinh). 2024 Apr 24;11(25):2401455. doi: 10.1002/advs.202401455 (PMC11220683; doi:10.1002/advs.202401455)
Supplement: Supplementary file 1 — Supporting Information [file ADVS-11-2401455-s001.pdf]

ADVANCED SCIENCE
Open Access

Supporting Information

for *Adv. Sci.*, DOI 10.1002/advs.202401455

Iron Active Center Coordination Reconstruction in Iron Carbide Modified on Porous Carbon for Superior Overall Water Splitting

Wenxin Guo, Jinlong Li, Dong-Feng Chai, Dongxuan Guo, Guozhe Sui, Yue Li, Dan Luo* and Lichao Tan**

© Copyright 2022. WILEY-VCH GmbH.

Supporting Information

Iron Active Center Coordination Reconstruction in Iron Carbide Modified on Porous Carbon for Superior Overall Water Splitting

Wenxin Guo[#], Jinlong Li[#], Dong-Feng Chai[#], Dongxuan Guo, Guozhe Sui, Yue Li, Dan Luo* and Lichao Tan**

W. Guo, Prof. J. Li, Prof. D. Guo, Prof. D.-F. Chai, Prof. G. Sui
College of Chemistry and Chemical Engineering, Key Laboratory of Fine Chemicals of College of Heilongjiang Province, Qiqihar University, Qiqihar 161006, China
E-mail: dongxuanguo92@gmail.com

Prof. L. Tan
Institute of Carbon Neutrality, Zhejiang Wanli University, Ningbo 315100, China
E-mail: tanlcking@163.com

Prof. Y. Li
School of Polymer Science & Engineering, Qingdao University of Science & Technology, Qingdao 266000, China

Prof. D. Luo
Department of Chemical Engineering, University of Waterloo, Waterloo, ON N2L 3G1, Canada
E-mail: luodan@dicp.ac.cn

Contents

Material Characterizations ······ S4

Electrochemical Measurements ······ S5

Theoretical section ······ S6

Figure S1. SEM and TEM images of Fe_3C/NC-350. ······ S7

Figure S2. SEM and EDS mapping of Fe_3C/NC-550. ······ S8

Figure S3. XRD spectrum of Fe_3C/NC-550, Fe_3C/NC-650, and Fe_3C/NC-750. ······ S9

Figure S4. XRD spectrum of elm seeds-derived N-droped carbon loaded Fe_3C nanoparticles. ········ S10

Figure S5. XRD spectrum of corn leaves-derived N-droped carbon loaded Fe_3C nanoparticles.. ········ S11

Figure S6. XRD spectrum of shaddock peel-derived N-droped carbon loaded Fe_3C nanoparticles.········ S12

Figure S7. XPS survey spectrum of Fe_3C/NC, Fe_3C/NC-350 and Fe_3C/NC-550.············ S13

Figure S8. The Fe_3C/NC, Fe_3C/NC-350 and Fe_3C/NC-550 plots the markedly different k space oscil-lation. ········ S15

Figure S9. The wavelet transform (WT) EXAFS contour map of Fe foil and Fe_2O_3. ······· S15

Figure S10. LSV curves of Fe_3C/NC-550, Fe_3C/NC-650 and Fe_3C/NC-750 for HER. ····· S16

Figure S11. The LSV curves of Fe_3C/NC, Fe_3C/NC-350 and Fe_3C/NC-550 for elm seeds, corn leaves and shaddock peel. ········ S17

Figure S12. LSV curves of as-obtained catalysts after normalizing the current by ECSA. · S18

Figure S13 . The i-t curve of Fe_3C/NC-550 for HER at -100 mA/cm^2········ S19

Figure S14 . SEM image of Fe_3C/NC-550 after testing ········ S20

Figure S15. XRD spectrum of Fe_3C/NC-550 and the sample after cycling test. ············ S21

Figure S16. XPS spectrum of Fe_3C/NC-550 after testing. ········ S22

Figure S17. The amount of H_2 and O_2 produced by the cathode and anode in 1 M KOH solution (a); The process of overall water splitting (b). ········ S23

Figure S18. The HER chemisorption processes of Fe_3C/NC-550.········ S24

Figure S19. The OER chemisorption processes of Fe_3C/NC-550.········ S25

Figure S20. The d-band center of Fe_3C/NC, Fe_3C/NC-350 and Fe_3C/NC-550. ············· S26

Table S1. Fe *K*-edge EXAFS least-squares fitting parameters[a] for Fe foil, FeO, Fe_2O_3, Fe_3C/NC, Fe_3C/NC-350 and Fe_3C/NC-550 sample. ·· S27

References·· S29

Material Characterizations: X-ray diffraction (XRD) measurements were implemented by a powder X-ray diffraction system (Rigaku, TTR-III) equipped with Cu Kα radiation (λ=0.15406 nm) to identify structures of the obtained composites. The X-ray photoelectron spectroscopy (XPS) measurements were implemented by a Thermo ESCALAB 250Xi spectrometer with monochromatic Al Kα radiation (hγ=1486.6 eV). All XPS spectra were characterized with respect to the C 1s peak at 284.6 eV. The structure of the as-fabricated products was investigated by scanning electron microscope (FE-SEM) (Hitachi, SU8000) and a transmission electron microscopy (TEM) (JEOL, JEM-2010, 200 kV). EPR tests were carried out in the X-band (9.45 GHz) with 5.00-G modulation amplitude and a magnetic field modulation of 100 kHz using a Bruker EPR spectrometer (A300-10-12, Bruker) at 77 K. Nitrogen adsorption-desorption experiments were carried out at 77.35 K by means of an Autosorb-1 (Quantachrome Instruments) analyzer. The X-ray absorption fine structures (XAFSs), including X-ray absorption near-edge structures (XANESs) and extended X-ray absorption fine structures (EXAFSs) Data reduction, data analysis, and EXAFS fitting were performed with the Athena and Artemis software packages.[1, 2] The energy calibration of the sample was conducted through a standard Ni foil, which as a reference was simultaneously measured. For EXAFS modeling, EXAFS of the Ni foil is fitted and the obtained amplitude reduction factor S02 value (0.795) was set Ni the EXAFS analysis to determine the coordination numbers (CNs) Ni the Ni-Ni scattering paths Ni sample. Data reduction, data analysis, and EXAFS fitting were performed with the Athena and Artemis software packages. The energy

calibration of the sample was conducted through a standard Fe foil, which as a reference was simultaneously measured. For EXAFS modeling, EXAFS of the Fe foil is fitted and the obtained amplitude reduction factor S02 value (0.760) was set Fe the EXAFS analysis to determine the coordination numbers (CNs) Fe the Fe-Fe scattering paths Fe sample.

Electrochemical Measurements: All HER electrochemical performance tests were performed in 1 M KOH (pH=14) solution. The working electrode, reference electrode, and counter electrode are self-supporting electrode carrying electrocatalysts (1 cm×1 cm), saturated calomel electrode and graphite rods, respectively. Before the linear scanning voltammetry (LSV) test, the electrocatalyst needs to be activated. Cyclic voltammetry (CV) technology is used and the scan rate is 100 mV/s until a stable CV curve appears. The linear sweep voltammetry test has a sweep speed of 5 mV/s.

To measure electrochemical double-layer capacitance (C_{dl}), the potentials were swept for a cycle using RDE at 1,600 rpm. at a range of no faradic processes six times at six different scan rates. The measured capacitive current densities at the average potential in the selected range were plotted as a function of the scan rates and the slope of the linear fit could be calculated as the C_{dl}.

Theoretical section: We have employed the first-principles[3, 4] to perform all density functional theory (DFT) calculations within the generalized gradient approximation (GGA) using the Perdew-Burke-Ernzerhof (PBE)[5] formulation. We have chosen the projected augmented wave (PAW) potentials[6, 7] to describe the ionic cores and take valence electrons into account using a plane wave basis set with a kinetic energy cutoff of 520 eV. Partial occupancies of the Kohn−Sham orbitals were allowed using the Gaussian smearing method and a width of 0.05 eV. The electronic energy was considered self-consistent when the energy change was smaller than 10^{-4} eV. A geometry optimization was considered convergent when

the energy change was smaller than 0.05 eV $Å^{-1}$. In our structure, the U correction is used for Fe and Ni atoms. The vacuum spacing in a direction perpendicular to the plane of the structure is 20 Å for the surfaces. The Brillouin zone integration is performed using 2×2×1 Monkhorst-Pack k-point sampling for a structure. Finally, the adsorption energies (Eads) were calculated as Eads= Ead/sub-Ead-Esub, where Ead/sub, Ead, and Esub are the total energies of the optimized adsorbate/substrate system, the adsorbate in the structure, and the clean substrate, respectively. The free energy was calculated using the equation:

$$G=Eads+ZPE-TS$$

Where G, Eads, ZPE and TS are the free energy, total energy from DFT calculations, zero point energy and entropic contributions, respectively.

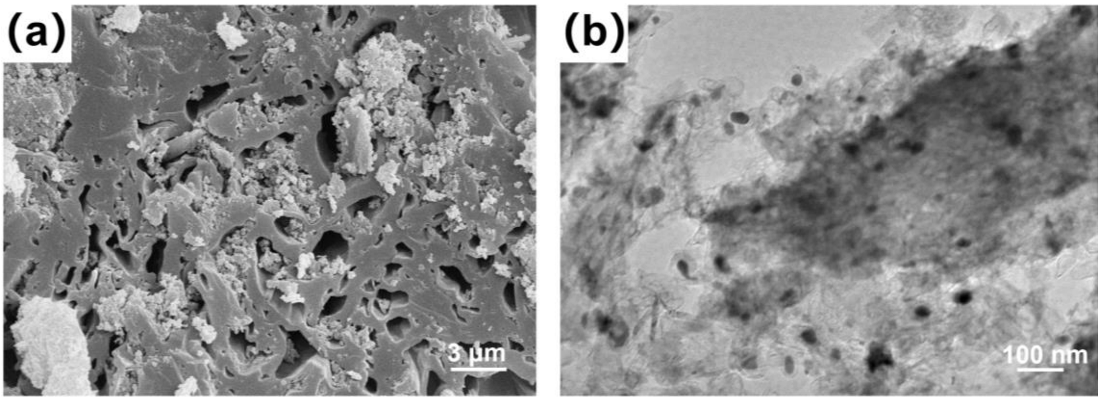

Figure S1. SEM and TEM images of Fe_3C/NC-350.

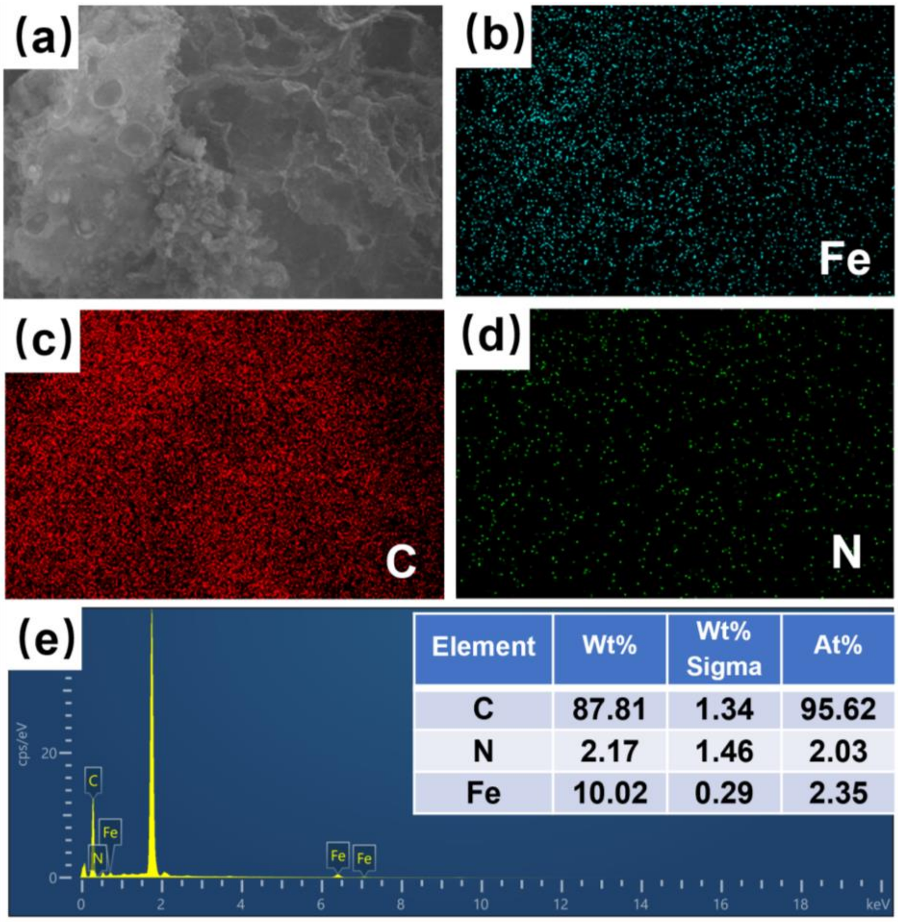

Element	Wt%	Wt% Sigma	At%
C	87.81	1.34	95.62
N	2.17	1.46	2.03
Fe	10.02	0.29	2.35

Figure S2. SEM and EDS mapping of Fe_3C/NC-550.

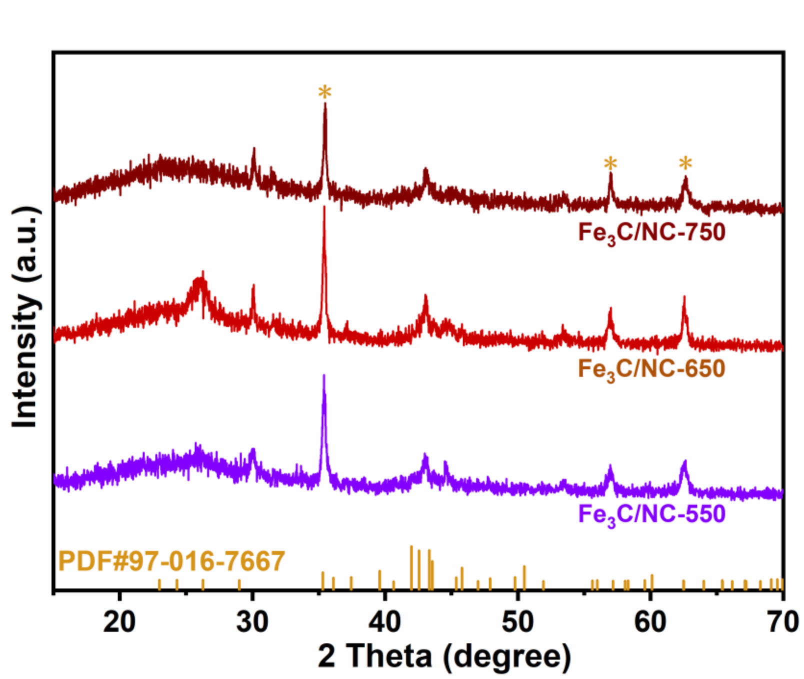

Figure S3. XRD spectrum of Fe_3C/NC-550, Fe_3C/NC-650, and Fe_3C/NC-750.

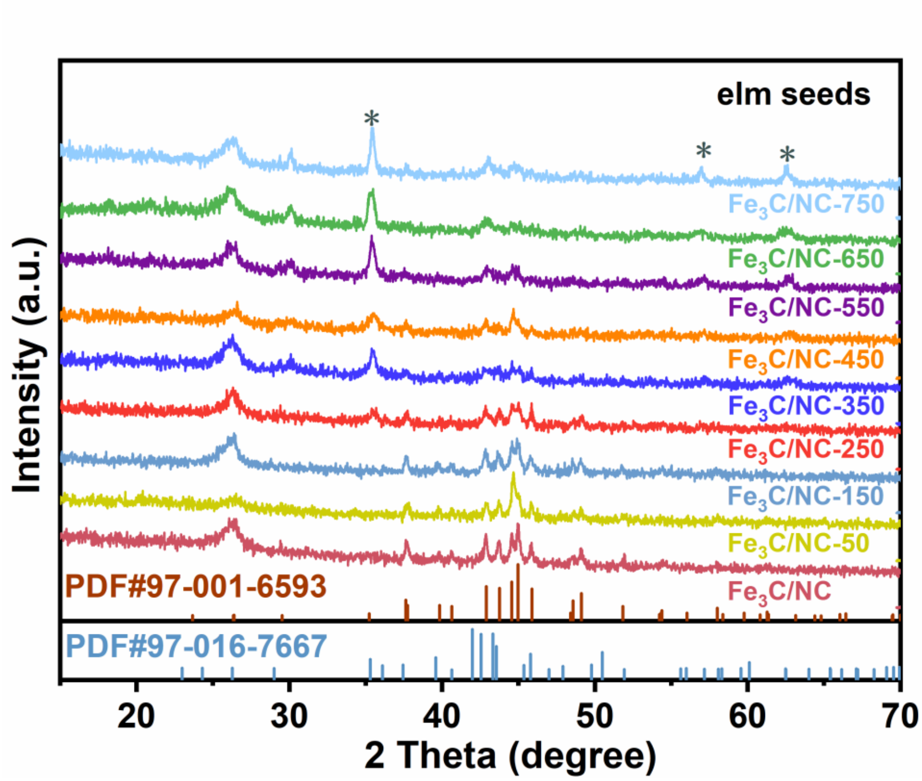

Figure S4. XRD spectrum of elm seeds-derived N-droped carbon loaded on Fe_3C nanoparticles.

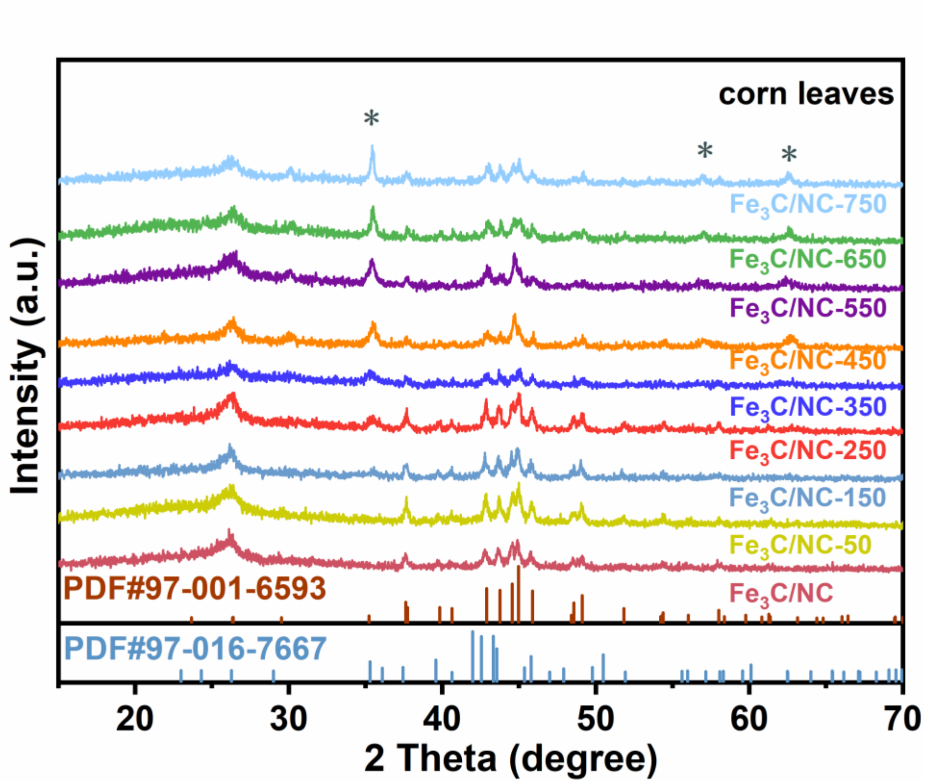

Figure S5. XRD spectrum of corn leaves-derived N-droped carbon loaded on Fe_3C nanoparticles.

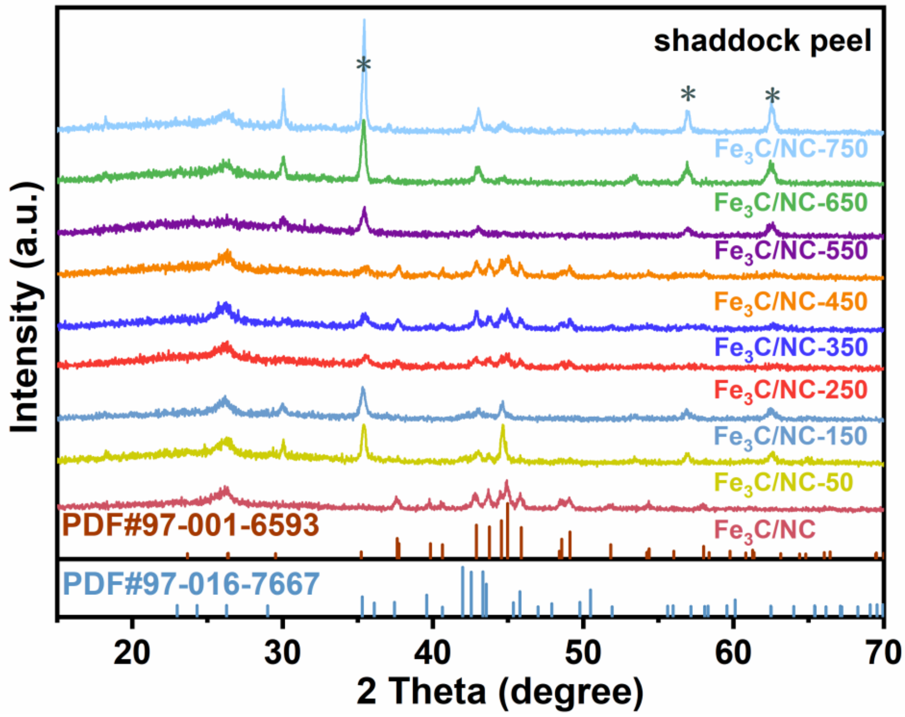

Figure S6. XRD spectrum of shaddock peel-derived N-droped carbon loaded on Fe_3C nanoparticles.

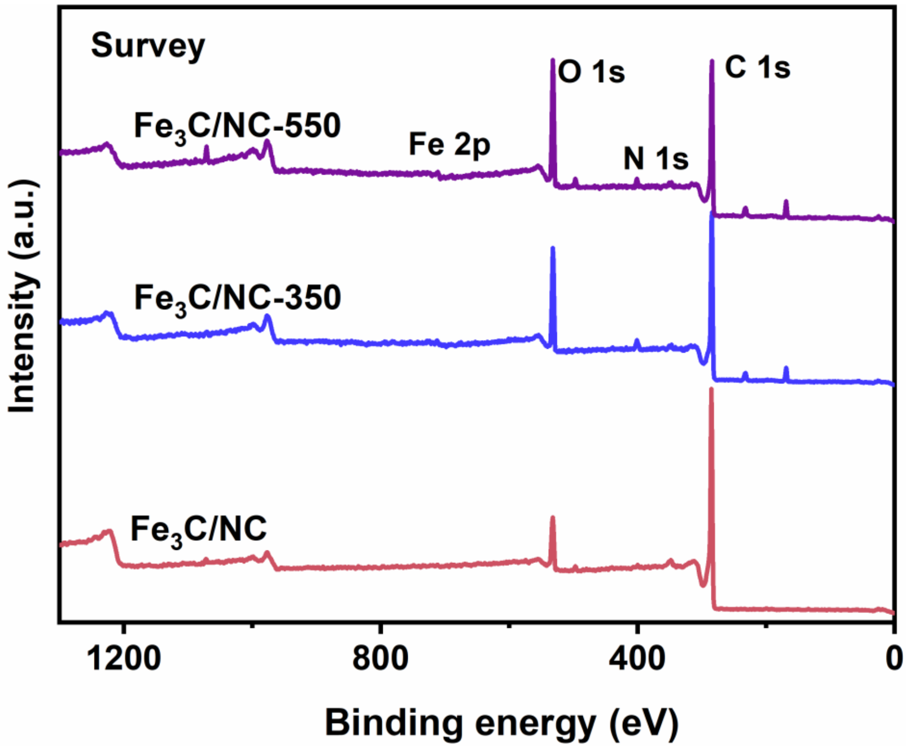

Figure S7. XPS survey spectrum of Fe_3C/NC, Fe_3C/NC-350 and Fe_3C/NC-550.

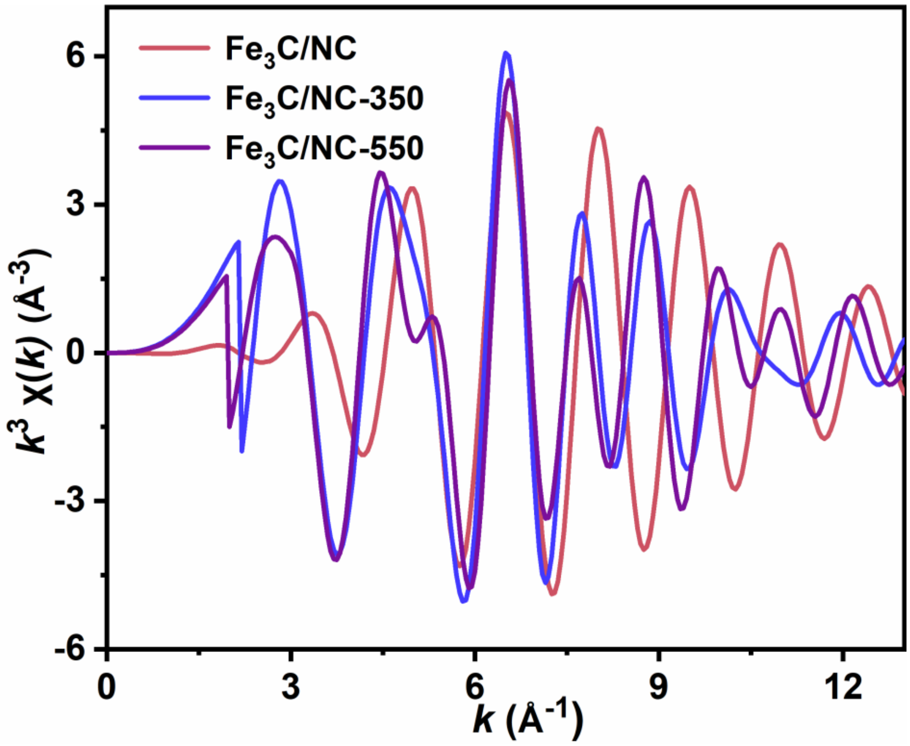

Figure S8. The Fe_3C/NC, Fe_3C/NC-350 and Fe_3C/NC-550 plots the markedly different k space oscil-lation.

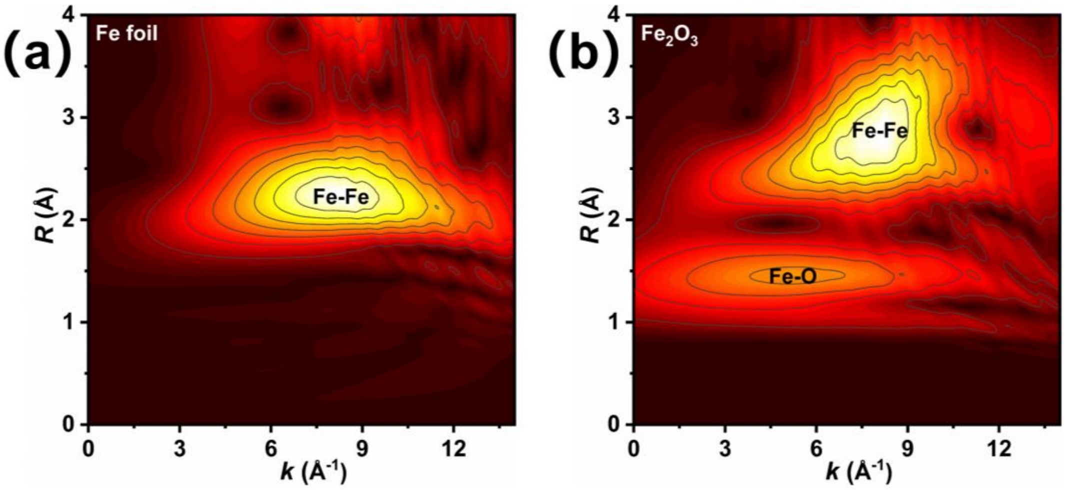

Figure S9. The wavelet transform (WT) EXAFS contour map of Fe foil and Fe_2O_3.

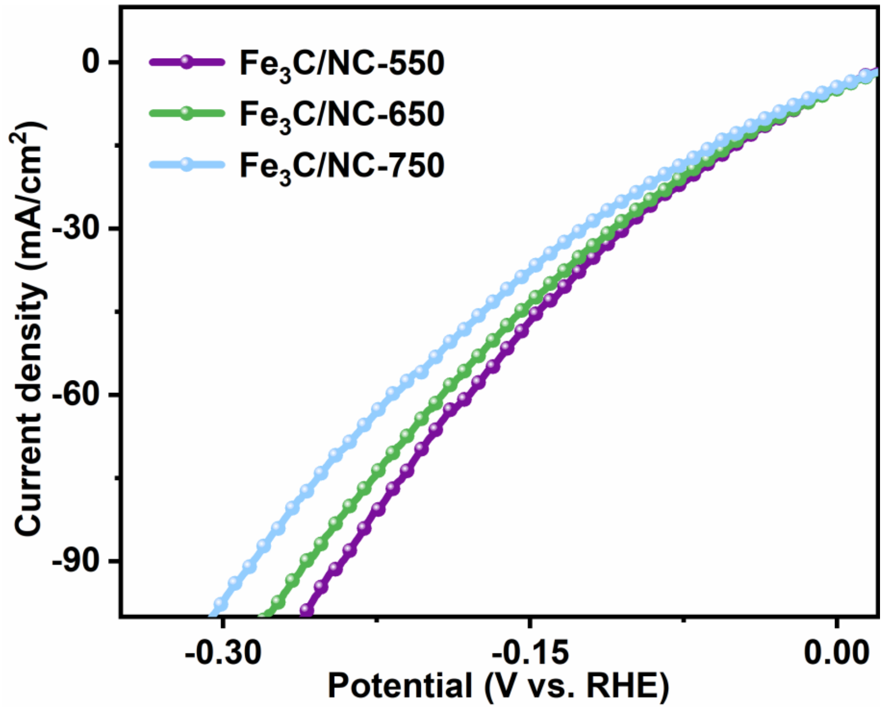

Figure S10. LSV curves of Fe_3C/NC-550, Fe_3C/NC-650 and Fe_3C/NC-750 for HER.

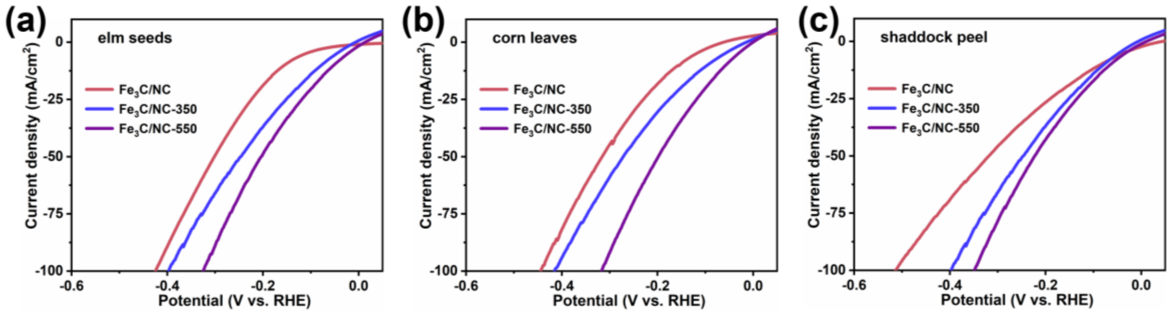

Figure S11. The LSV curves of Fe_3C/NC, Fe_3C/NC-350 and Fe_3C/NC-550 for elm seeds (a), corn leaves (b) and shaddock peel (c).

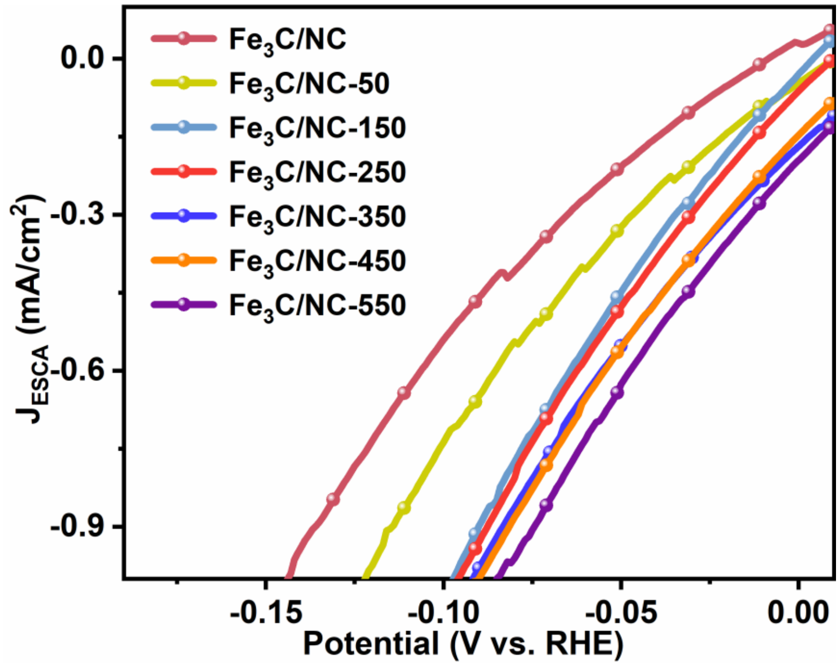

Figure S12. LSV curves of as-obtained catalysts after normalizing the current by ECSA.

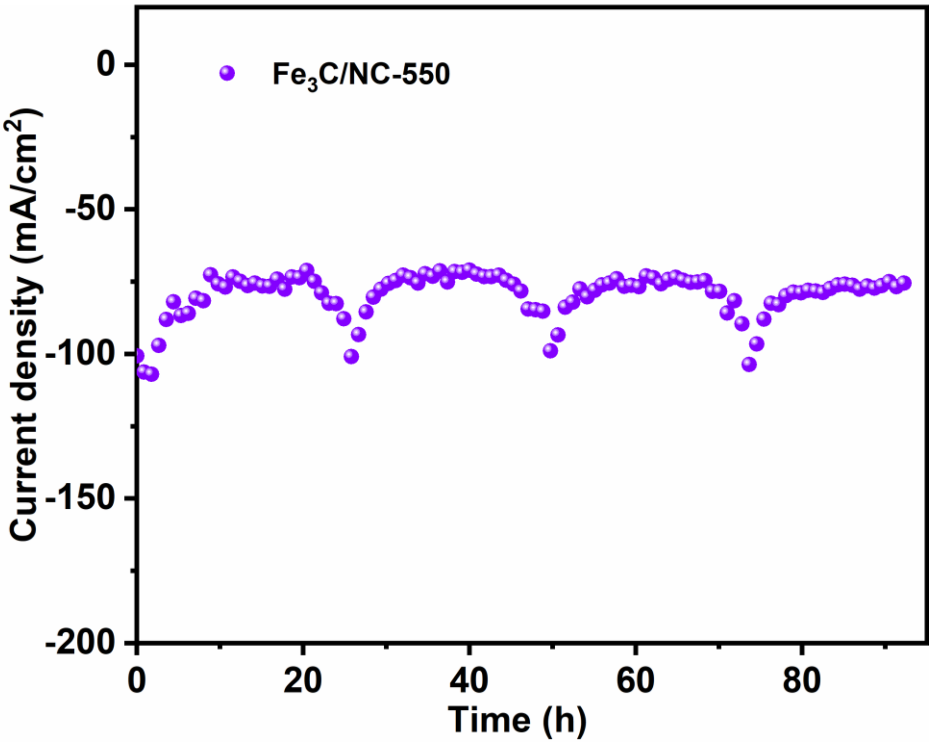

Figure S13. The i-t curve of Fe_3C/NC-550 for HER at -100 mA/cm^2.

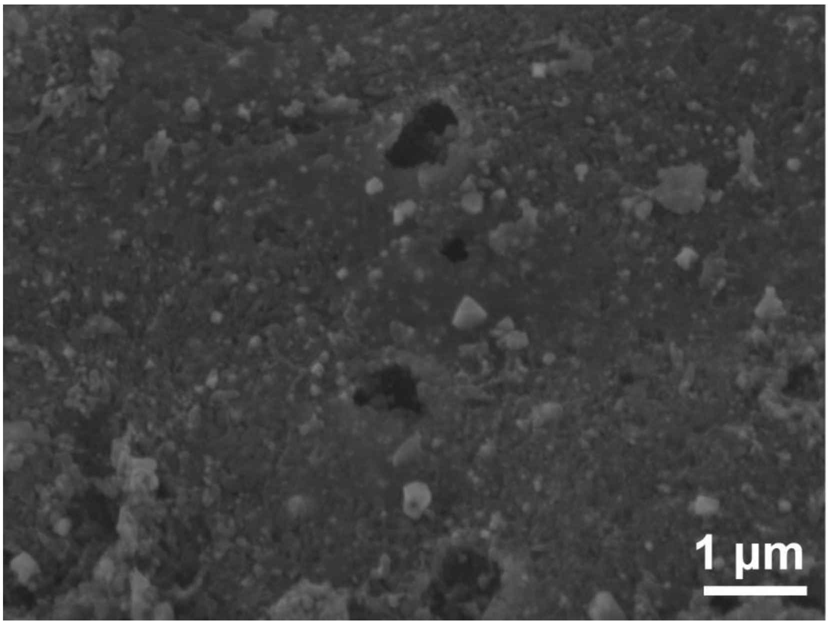

Figure S14. SEM image of Fe_3C/NC-550 after testing.

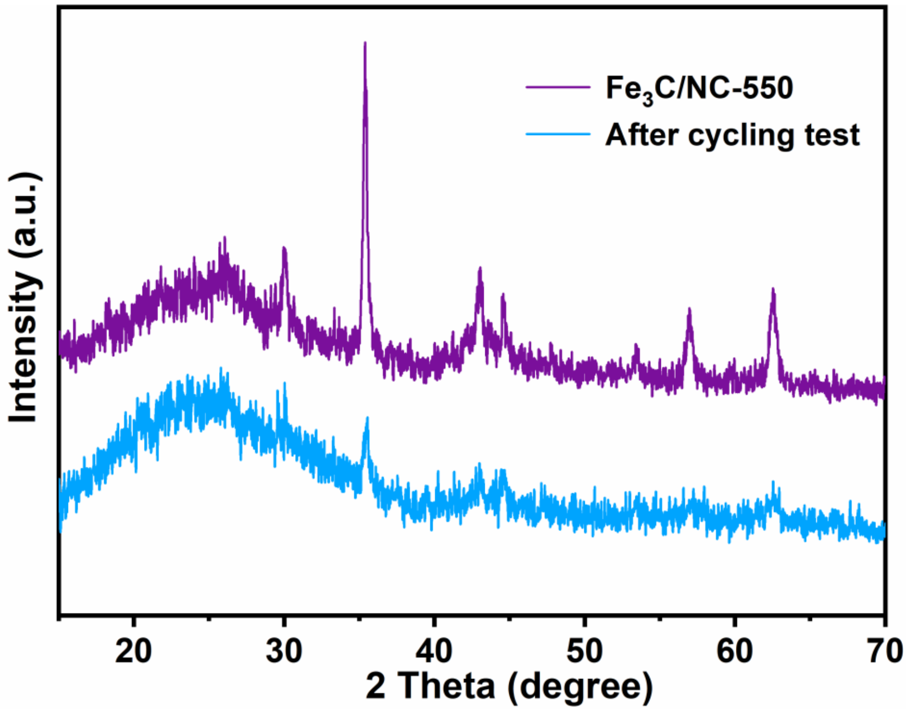

Figure S15. XRD spectrum of Fe_3C/NC-550 and the sample after cycling test.

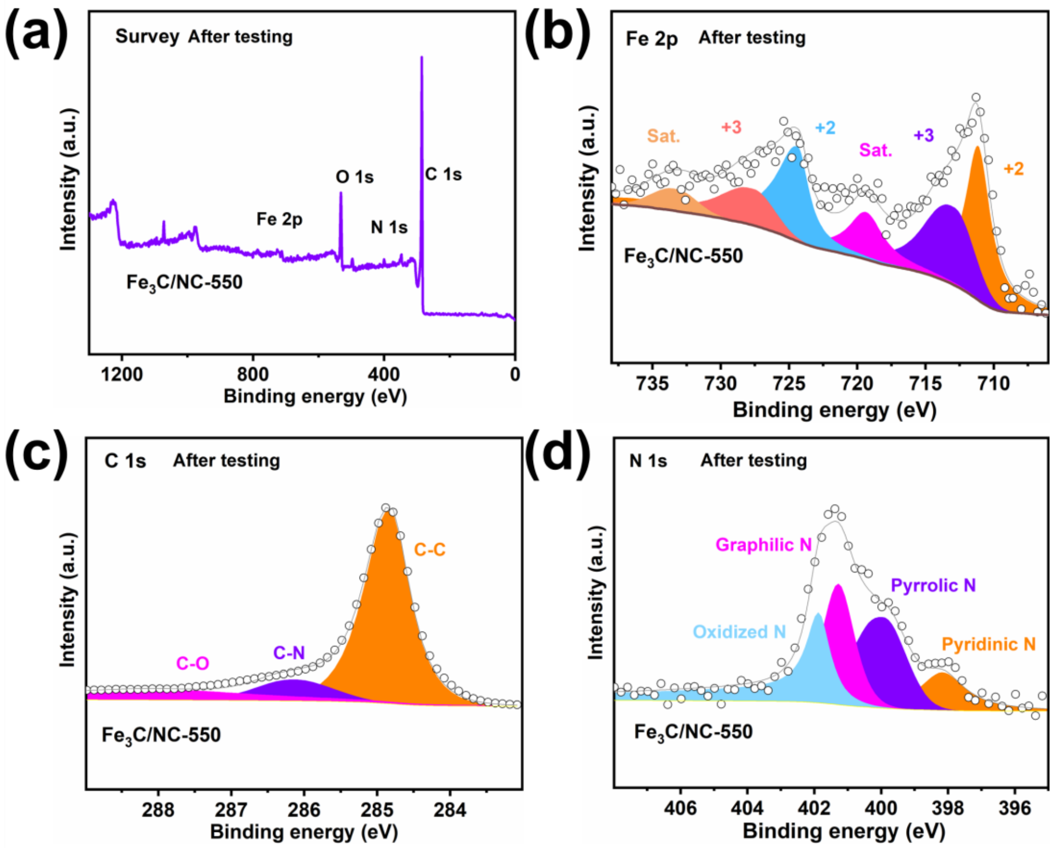

Figure S16. XPS spectrum of Fe_3C/NC-550 after testing.

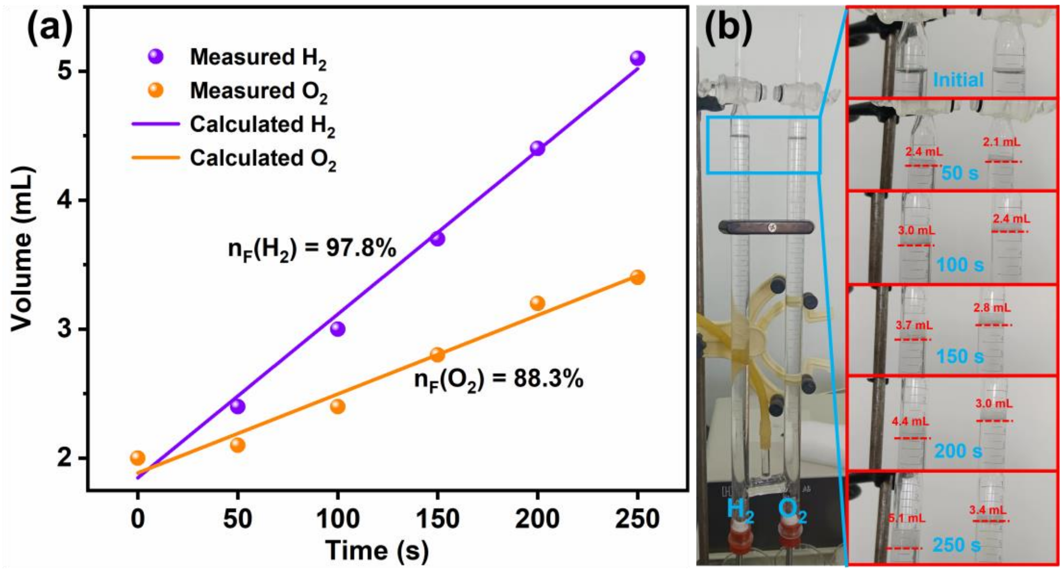

Figure S17. The amount of H_2 and O_2 produced by cathode and anode in 1.0 M KOH solution (a). The process of overall water splitting (b).

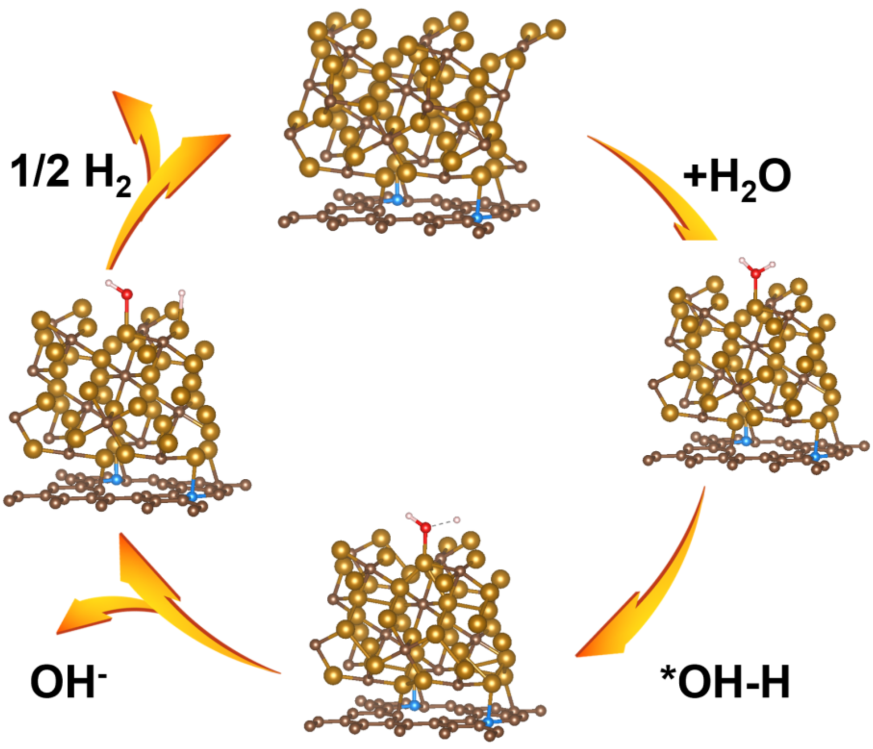

Figure S18. The HER chemisorption processes of Fe_3C/NC-550.

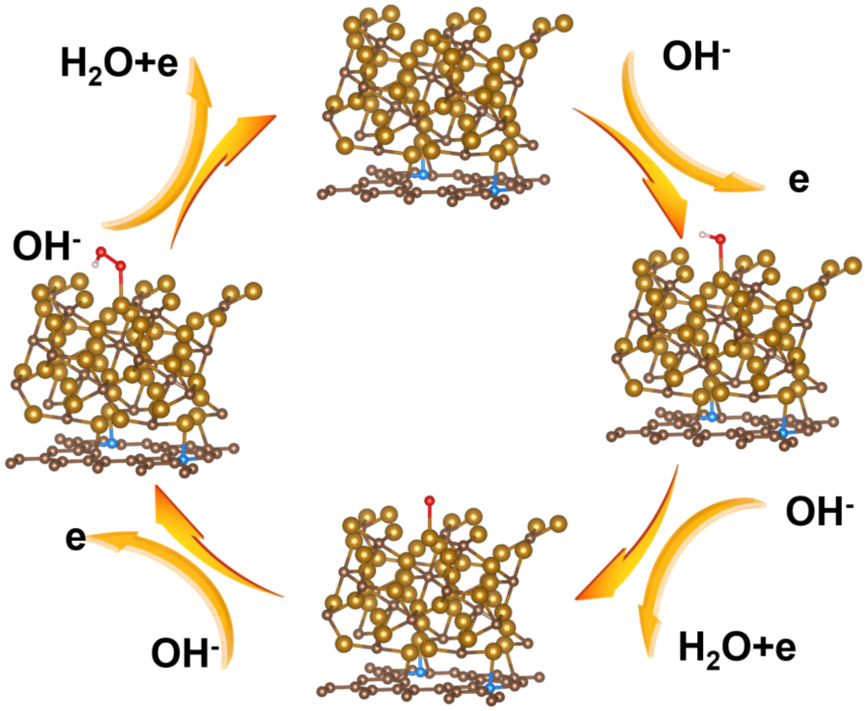

Figure S19. The OER chemisorption processes of Fe_3C/NC-550.

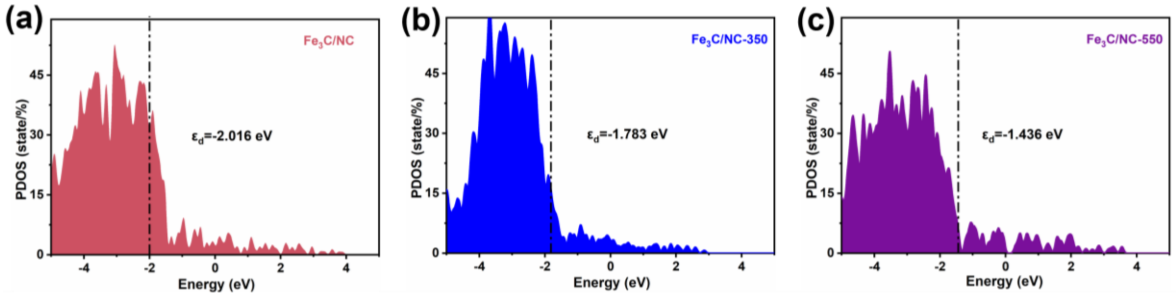

Figure S20. The d-band center of Fe_3C/NC (a), Fe_3C/NC-350 (b) and Fe_3C/NC-550 (c).

Table S1. Fe *K*-edge EXAFS least-squares fitting parameters[a] for Fe foil, FeO, Fe_2O_3 standard, and Fe samples.

**Material**	**Path**	***N***	***R*** (Å)	σ^2 (Å^2)	**Δ*E*0** (eV)	***R*-factor**
**Fe foil**[b]	Fe-Fe1	8	2.468±0.018	0.00343	6.48±0.37	0.413%
	Fe-Fe2	6	2.853±0.017	0.00407		
**FeO**[c]	Fe-O	6	2.123±0.033	0.01559	2.32±0.26	0.812%
	Fe-Fe	12	3.071±0.023	0.01388		
**Fe_2O_3**[d]	Fe-O1	3	1.958±0.022	0.00229	0.38±0.65	0.815
	Fe-O2	3	2.123±0.004	0.00654		
**Fe_3C/NC**[e]	Fe-Fe	**3.32±0.12**	**2.456±0.023**	0.00751	-4.00±0.39	0.558%
**Fe_3C/NC-350**[f]	Fe-C	**3.04±0.17**	**2.023±0.064**	0.00936	5.13±0.47	0.927%
	Fe-Fe1	**1.82±0.23**	**2.640±0.026**	0.00880		
	Fe-Fe2	**2.26±0.33**	**3.080±0.020**	0.00880		
**Fe_3C/NC-550**[g]	Fe-Fe	**3.96±0.38**	**3.063±0.036**	0.00936	5.16±0.52	2.10%
	Fe-C	**2.63±0.19**	**1.991±0.096**	0.00729		

[a]*N*, coordination number; *R*, distance between absorber and backscatter atoms; σ^2, Debye-Waller factor to account for both thermal and structural disorders; ΔE_0, inner potential correction; *R*-factor (%) generally estimates the goodness of the fit. Error bounds (accuracies) that characterize the structural parameters obtained by EXAFS spectroscopy were estimated as *N*±20%; *R*±1%; σ^2±20%; ΔE_0±20%. S_0^2 was fixed as 0.78, which was determined by fitting the experimental data on Fe foil with fixed coordination numbers (in bold) according to the crystal structure, as well for FeO and Fe_2O_3 references. [b] Fitting range for Fe foil was selected to be $3.5 \leq k \leq 12.2$ Å^{-1} (k^3-weighted) and $1.3 \leq R \leq 3.0$ Å, yielding the number of variable parameters being 4, out of a total of 9.12 independent data points. [c] Fitting range for FeO was selected to

be 3.1 ≤ k ≤ 11.4 $Å^{-1}$ (k^3-weighted) and 1.3 ≤ R ≤ 3.3 Å, yielding the number of variable parameters being 3, out of a total of 10.31 independent data points. [d] Fitting range for Fe_2O_3 was selected to be 1.8 ≤ k ≤ 10.6 $Å^{-1}$ (k^3-weighted) and 1.0 ≤ R ≤ 2.1 Å, yielding the number of variable parameters being 3, out of a total of 5.98 independent data points. [e]Fitting range for 764-1-Fe sample was selected to be 3.0 ≤ k ≤ 8.0 $Å^{-1}$ (k^3-weighted) and 1.0 ≤ R ≤ 3.0 Å, yielding the number of variable parameters being 2, out of a total of 6.19 independent data points; [f]Fitting range for 764-2-Fe sample was selected to be 3.5 ≤ k ≤ 9.0 $Å^{-1}$ (k^3-weighted) and 1.0 ≤ R ≤ 3.5 Å, yielding the number of variable parameters being 4, out of a total of 8.62 independent data points; [g]Fitting range for 764-3-Fe sample was selected to be 3.5 ≤ k ≤ 9.0 $Å^{-1}$ (k^3-weighted) and 1.0 ≤ R ≤ 3.8 Å, yielding the number of variable parameters being 3, out of a total of 9.58 independent data points.

References

[1] B. Ravel, M. Newville, ATHENA, ARTEMIS, HEPHAESTUS: data analysis for X-ray absorption spectroscopy using IFEFFIT, *J. Synchrotron. Radiat.* **2005**, *12*, 537.

[2] H. Funke, A.C. Scheinost, M. Chukalina, Wavelet analysis of extended x-ray absorption fine structure data, *Phys. Rev. B* **2005**, *71*, 232.

[3] Kresse, G.; Furthmüller, J. Efficiency of Ab-Initio total energy calculations for metals and semiconductors using a plane-wave basis set. *Comput. Mater. Sci.* **1996**, *6*, 15.

[4] Kresse, G.; Furthmüller, J. Efficient iterative schemes for Ab initio total-energy calculations using a plane-wave basis Set. *Phys. Rev. B* **1996**, *54*, 11169.

[5] Perdew, J. P.; Burke, K.; Ernzerhof, M. Generalized gradient approximation made simple. *Phys. Rev. Lett.* **1996**, *77*, 3865.

[6] Kresse, G.; Joubert, D. From ultrasoft pseudopotentials to the projector augmented-wave

method. *Phys. Rev. B* **1999**, *59*, 1758.

[7] P.E. Blochl, Projector augmented-wave method, *Phys. Rev. B Condens. Matter.* **1994**, *50*, 17953.
